# Extração de Dispositivos Venosos Centrais Totalmente Implantáveis pela Técnica PISA: Uma Pequena Série de Casos

**DOI:** 10.36660/abc.20250569

**Published:** 2026-07-22

**Authors:** Margarida G. Figueiredo, Hélder Santos, Bruno Valente, Mário Oliveira

**Affiliations:** 1 Hospital de Santa Marta Unidade de Arritmologia, Estimulação Cardíaca e Eletrofisiologia Lisboa Portugal Hospital de Santa Marta – Unidade de Arritmologia, Estimulação Cardíaca e Eletrofisiologia, Lisboa – Portugal; 2 Universidade de Lisboa Faculdade de Medicina Lisboa Portugal Faculdade de Medicina, Universidade de Lisboa, Lisboa – Portugal; 3 Comprehensive Health Research Center Lisboa Portugal Comprehensive Health Research Center (CHRC), Lisboa – Portugal

**Keywords:** Equipamentos e Suprimentos, Cateteres Venosos Centrais, Relatos de Casos

## Introdução

O progresso tecnológico levou ao uso generalizado de dispositivos médicos implantáveis em diversas condições, melhorando significativamente os resultados para os pacientes. Em oncologia, os cateteres venosos centrais totalmente implantáveis (CVCTI) são comumente usados para facilitar a administração prolongada de quimioterapia. O término da terapia é a indicação mais comum para a remoção. No entanto, diferentes complicações associadas ao uso desses dispositivos foram descritas,^1^ e algumas delas podem justificar sua remoção.^2^ Embora historicamente realizada por cirurgiões, a remoção de CVCTI por radiologia intervencionista tornou-se muito mais frequente nas últimas décadas. A remoção desses dispositivos pode se tornar tecnicamente desafiadora quando se desenvolve uma aderência fibrótica significativa ao redor da ponta do cateter ou ao longo de seu trajeto intravascular.^3^

A técnica PISA,^4^ originalmente desenvolvida para extrair eletrodos de dispositivos eletrônicos cardíacos implantáveis, pode ser adaptada para a remoção de cateteres venosos centrais implantáveis. Ela envolve a identificação e exposição da porção proximal do cateter, seguida de dissecção até o ponto de entrada vascular. Uma bainha de polipropileno é então avançada sobre o cateter usando movimento rotacional e tração leve para liberar aderências e obter a extração completa.^5^

Considerando a segurança e a relação custo-benefício associadas à técnica de extração PISA, esta Carta de Pesquisa tem como objetivo descrever seus princípios subjacentes e aplicação prática, destacando seu potencial como método alternativo para a remoção desses dispositivos.

## Paciente 1

**Figura 1:**
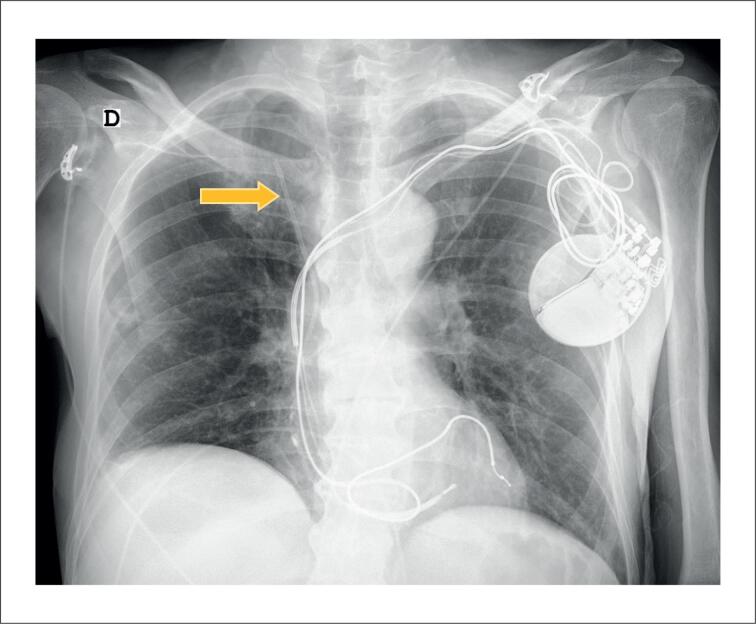
Radiografia torácica da porção distal do cateter venoso central abandonado (seta amarela) e do TRC-D com eletrodo de desfibrilação de bobina dupla.

Um homem de 60 anos com histórico de cardiomiopatia dilatada teve um desfibrilador de terapia de ressincronização cardíaca (TRC-D) implantado em 2009. Recentemente, no contexto de um câncer colorretal recém-diagnosticado, ele foi submetido a uma ileostomia de derivação, que levou ao desenvolvimento de síndrome do intestino curto, exigindo múltiplas intervenções cirúrgicas e internações hospitalares. Para a administração de quimioterapia, foi inserido um CVCTI. Em um ano, a suspeita de infecção do CVCTI levou à sua remoção cirúrgica, mas o dispositivo fraturou durante o procedimento, permitindo a remoção apenas do reservatório do Port-a-Cath, deixando o cateter distal irrecuperável apesar de múltiplas tentativas (Figura 1); um novo CVCTI femoral direito foi implantado posteriormente. Seis meses depois, o paciente desenvolveu uma síndrome de emagrecimento acompanhada de febre. As hemoculturas foram positivas para *Staphylococcus aureus* resistente à meticilina. Um ecocardiograma transesofágico (ETE) revelou endocardite infecciosa associada ao eletrodo do TRC-D. Além de iniciar antibioticoterapia com rifampicina e daptomicina, foi realizada a extração percutânea de todos os dispositivos intravasculares. A extração dos eletrodos foi realizada utilizando a abordagem PISA via veia axilar esquerda, e a remoção completa dos três eletrodos foi alcançada. Como o acesso femoral direito era necessário para a remoção do cateter retido — e considerando o risco infeccioso associado — o CVCTI femoral direito foi removido. Por fim, utilizando um laço introduzido pela veia femoral direita, o fragmento retido do cateter subclávio direito foi removido com sucesso. O procedimento transcorreu sem intercorrências, sem complicações pós-operatórias imediatas. O paciente foi transferido de volta ao hospital de origem no dia seguinte para completar o ciclo planejado de seis semanas de antibióticos intravenosos para infecção ativa relacionada ao dispositivo.

## Paciente 2

**Figura 2:**
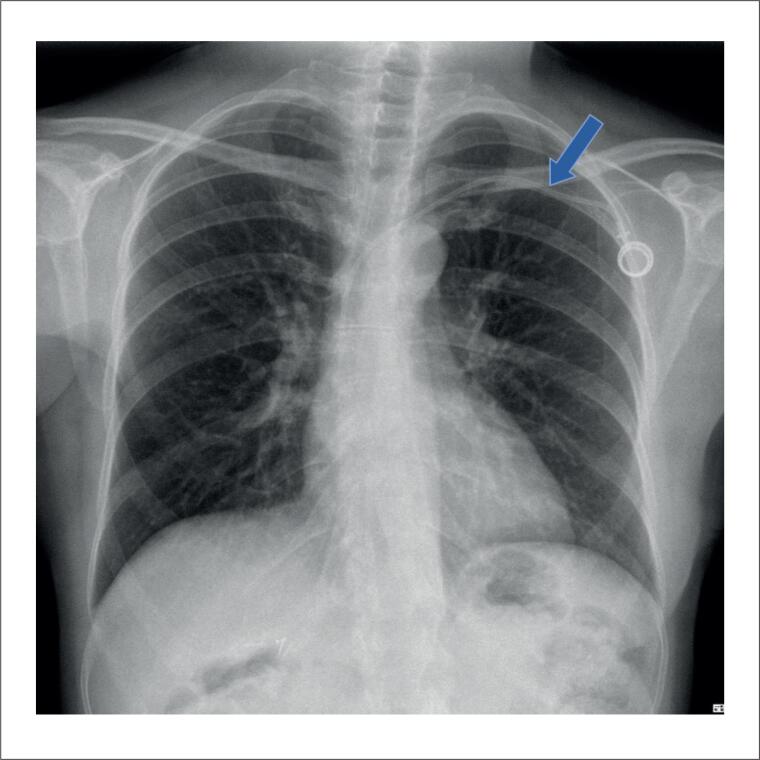
Radiografia torácica do CVCTI (seta azul).

Uma mulher de 58 anos com histórico de câncer de mama direito diagnosticado em 2009 foi submetida a quimioterapia neoadjuvante e adjuvante, para a qual foi inserido um CVCTI na veia subclávia esquerda em 2010. A paciente permaneceu em acompanhamento oncológico por mais de dez anos, sem evidência de recidiva. Durante esse período, foi detectada uma falha no dispositivo, e não foi possível realizar a lavagem adequada do lúmen do cateter, o que implica um aumento exponencial de fenômenos embólicos, secundários ao ambiente pró-coagulante relacionado à adesão da fibrina ao cateter.^6^ Por esse motivo, a radiologia intervencionista tentou remover o CVCTI) não funcional, sem sucesso. A paciente foi então encaminhada ao departamento de cardiologia para extração percutânea eletiva do dispositivo venoso central (Figura 2). Durante o procedimento, a porção proximal do cateter foi exposta cirurgicamente e dissecada na loja do gerador, no lado esquerdo. Um fio-guia foi então introduzido através do lúmen pérvio do dispositivo e, utilizando a abordagem PISA, a extração completa do cateter foi realizada com sucesso, tanto clínico quanto radiológico. A internação da paciente transcorreu sem intercorrências, e ela recebeu alta no dia seguinte.

## Paciente 3

**Figura 3:**
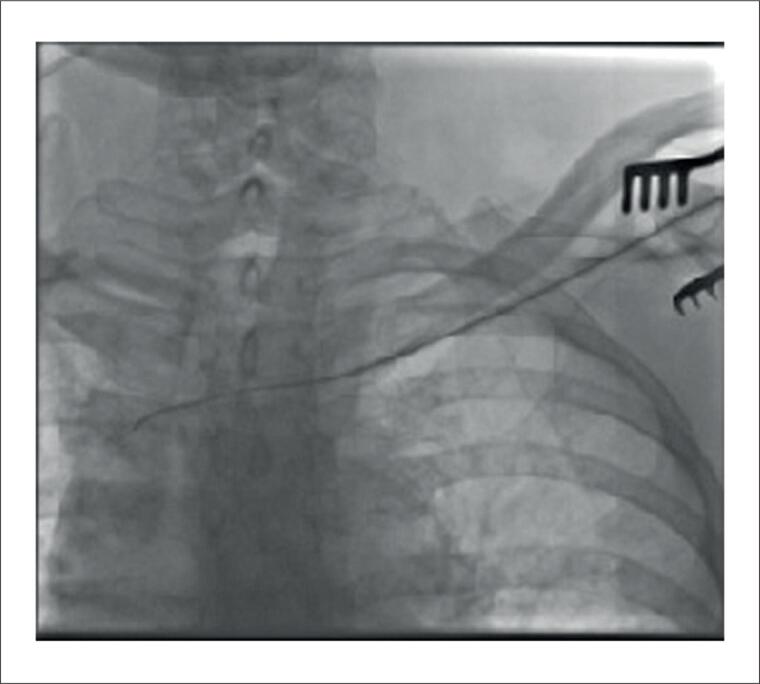
Radiografia torácica de um CVCTI com fio-guia em seu interior, para remoção do dispositivo utilizando a técnica PISA.

Uma mulher de 63 anos com histórico de hipotireoidismo, depressão e câncer de mama, submetida a mastectomia direita seis anos antes, seguida de seis ciclos de quimioterapia e terapia hormonal, e que, nesse contexto, tinha um CVCTI. Nos anos seguintes, foi documentada remissão total da patologia, deixando o dispositivo sem uso, apesar da necessidade de manutenção repetida para evitar complicações. Entretanto, durante esse período, foi constatada uma disfunção do CVCTI. Diante da ausência de qualquer indicação contínua para seu uso, foi realizada uma simples tentativa de tração para extrair completamente o dispositivo. Contudo, apenas o reservatório do Port-a-Cath pôde ser removido, enquanto o cateter permaneceu no mesmo local. Por esse motivo, a paciente foi encaminhada para extração da porção distal do cateter abandonado em nosso centro. Através da porção proximal do cateter, localizada no Port-a-Cath previamente removido, e utilizando bainhas de dilatação com a técnica PISA, o cateter foi completamente removido (Figuras 3 e 4). Não foram registradas complicações e a paciente recebeu alta no dia seguinte ao procedimento.

**Figura 4:**
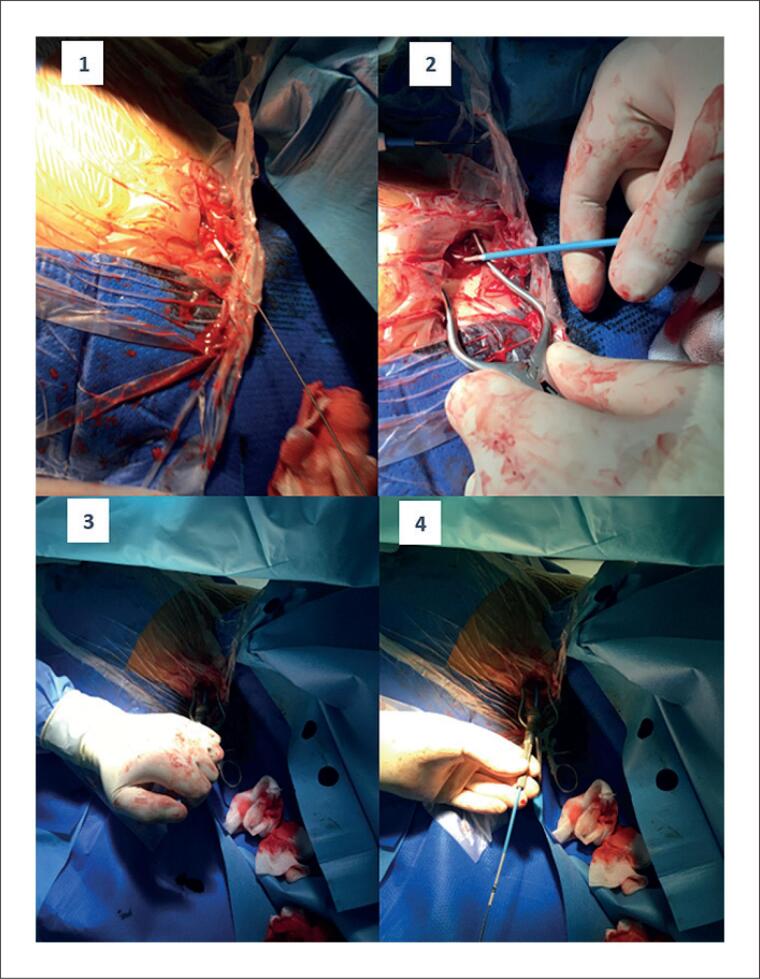
Imagens do procedimento de extração do CVCTI utilizando a técnica PISA.

## Paciente 4

Um homem de 75 anos com histórico de adenocarcinoma do cólon transverso foi submetido a ressecção cirúrgica um ano antes, seguida de oito ciclos de quimioterapia adjuvante, para os quais foi inserido um CVCTI.

Aos 10 meses de acompanhamento, foram identificadas metástases hepáticas e o paciente foi submetido a uma hepatectomia direita. O período pós-operatório foi complicado por diversos eventos, incluindo endocardite infecciosa, com isolamento de *Staphylococcus aureus* sensível à meticilina em hemoculturas. O ecocardiograma transesofágico confirmou a presença de vegetação e o paciente foi tratado com flucloxacilina intravenosa por quatro semanas, com melhora clínica.

Um mês depois, o paciente foi readmitido com choque séptico secundário à infecção por *Klebsiella pneumoniae* e tratado com antibioticoterapia direcionada. Dois dias após a alta, apresentou um novo episódio febril. Um novo ecocardiograma transesofágico (ETE) foi realizado, não evidenciando vegetações.

Considerando os episódios infecciosos recorrentes e a suspeita de foco infeccioso no CVCTI, o paciente foi encaminhado para extração do dispositivo. O procedimento foi realizado utilizando a técnica PISA sem complicações. Durante o acompanhamento de seis meses, não foram relatados novos episódios infecciosos.

## Discussão

A remoção CVCTIs de longa permanência é indicada quando esses dispositivos não são mais necessários ou quando ocorrem complicações ou mau funcionamento. Em casos de suspeita de infecção sistêmica, a infecção relacionada ao CVCTI deve sempre ser considerada e, se confirmada, a remoção completa do dispositivo é essencial.^5,7^

A técnica cirúrgica padrão para remoção de CVCTI envolve uma incisão sobre o reservatório, desprendimento do cateter, tração suave para extração e fechamento final da ferida. Embora as complicações durante a remoção sejam incomuns, o tempo de permanência prolongado está associado a maior dificuldade técnica devido ao desenvolvimento de uma bainha de fibrina ao redor do cateter, levando à aderência à túnica íntima venosa. Isso ocorre em aproximadamente 0,3–7,4% dos casos e está associado a uma maior probabilidade de necessidade de intervenções adicionais.^8^ A radiologia intervencionista oferece uma estratégia passo a passo para a remoção de cateteres aderentes, consistindo em três técnicas de complexidade crescente: suporte com fio-guia, separação coaxial anterógrada e separação coaxial retrógrada.^9^

Estudos anteriores demonstraram uma associação estatisticamente significativa entre a permanência do cateter por mais de 40 meses e a dificuldade de remoção.^10^ Com base nas evidências atuais, diversas recomendações foram propostas para o manejo seguro de CVCTIs: idealmente, esses dispositivos devem ser removidos logo após a conclusão do tratamento e, no máximo, cinco anos após a implantação, o que pode reduzir o risco de aderências intravasculares e encapsulamento fibrótico. Caso haja resistência durante a tentativa de extração, o procedimento deve ser interrompido imediatamente. A tração forçada aumenta o risco de fratura do cateter, avulsão vascular, tamponamento cardíaco, hemotórax e choque hemorrágico.^8^

Dentre as diversas abordagens para extração de dispositivos, a técnica PISA oferece uma alternativa segura e eficaz, particularmente em casos onde os métodos convencionais de extração não são bem-sucedidos. Em nosso centro, a técnica PISA é o método preferido para remoção de dispositivos por diversos motivos, incluindo a experiência consolidada do operador, maior controle e feedback tátil durante a manipulação do cateter, segurança do procedimento e custo-benefício em comparação com outras técnicas de extração. É importante ressaltar que essa abordagem também permite a interrupção segura do procedimento em qualquer etapa, caso os riscos superem os benefícios potenciais — uma consideração fundamental em indicações não infecciosas selecionadas.^5,11–13^ Essa abordagem utiliza bainhas de polipropileno (Cook Medical^®^) e bainhas de poliamida (FIAB^®^), selecionando-se a bainha cujo diâmetro interno seja o mais próximo possível do diâmetro do cateter para promover a dilatação e a ruptura de aderências fibróticas. Aplica-se torque controlado utilizando uma alça de dispositivo de torque (PIN VISE – Cook Medical^®^ ou MGB – FIAB^®^) para avançar a bainha mecânica, girando-a alternadamente no sentido horário e anti-horário, mantendo uma tração constante nas fitas de seda, liberando assim o eletrodo das aderências fibróticas circundantes. Este avanço rotacional facilita o deslocamento da ponta do eletrodo, permitindo a extração sem a necessidade de técnicas adicionais de extração.^5,11–16^

Um pré-requisito fundamental para esta técnica é que a extremidade proximal do cateter esteja acessível no local de entrada vascular; caso contrário, o procedimento não pode ser realizado. Este foi o caso do paciente 1, em quem o acesso à extremidade proximal não foi possível, sendo necessário o uso de um laço para extração. Em nossa série de casos, o uso da técnica PISA para extração de CVCTI foi clinicamente e radiologicamente bem-sucedido, mesmo após tentativas cirúrgicas prévias sem sucesso.

Até onde sabemos, este é o primeiro relato que descreve o uso da técnica PISA especificamente para a extração de CVCTI. A ausência de complicações e a alta taxa de sucesso observada sugerem que esse método pode ser uma opção valiosa em pacientes selecionados, particularmente aqueles com alto risco de infecção ou eventos embólicos. À medida que o uso de dispositivos de acesso venoso de longa duração continua a crescer, a adaptação de técnicas de extração já estabelecidas – como a abordagem PISA – a esses cateteres pode contribuir para melhores resultados em casos complexos. No entanto, estudos maiores serão necessários para confirmar esses achados.

## Data Availability

Os conteúdos subjacentes ao texto da pesquisa estão contidos no manuscrito.
