# Supplementary material for: Extração de Dispositivos Venosos Centrais Totalmente Implantáveis pela Técnica PISA: Uma Pequena Série de Casos
Source: Arq Bras Cardiol. 2026 Jul 22;123(7):e20250569. [Article in Portuguese] doi: 10.36660/abc.20250569 (PMC13399117; doi:10.36660/abc.20250569)
Supplement: Translation

# Translation: Extraction of Totally Implantable Venous Access Devices Using the PISA Technique: A Small Case Series

**DOI:** 10.36660/abc.20250569i

**Authors:** Margarida G. Figueiredo, Hélder Santos, Bruno Valente, Mário Oliveira

**Affiliations:** 1 Lisboa Portugal Hospital de Santa Marta – Unidade de Arritmologia, Estimulação Cardíaca e Eletrofisiologia, Lisboa – Portugal; 2 Lisboa Portugal Faculdade de Medicina, Universidade de Lisboa, Lisboa – Portugal; 3 Lisboa Portugal Comprehensive Health Research Center (CHRC), Lisboa – Portugal

**Keywords:** Equipment and Supplies, Central Venous Catheters, Case Reports

## Introduction

Technological progress has led to the widespread use of implantable medical devices across various conditions, significantly improving patient outcomes. In oncology, totally implantable central venous catheters (TICVCs) are commonly used to facilitate prolonged chemotherapy administration. The end of the therapy is the most common indication for removal. However, different complications associated with the use of these devices have been described,^1^ and some of them can justify their removal.^2^ Although historically performed by surgeons, removal of TICVC by interventional radiology has become much more frequent in recent decades. The removal of these devices can become technically challenging when significant fibrotic adhesion develops around the catheter tip or along its intravascular course.^3^

The PISA technique,^4^ originally developed for extracting leads from cardiac implantable electronic devices, can be adapted for TICVC removal. It involves identification and exposure of the catheter's proximal portion, followed by dissection to the vascular entry point. A polypropylene sheath is then advanced over the catheter using rotational motion and light traction to release adhesions and achieve complete extraction.^5^

Given the safety and cost-effectiveness associated with the PISA extraction technique, this Research Letter aims to describe its underlying principles and practical application, while highlighting its potential role as an alternative method for the removal of these devices.

## Patient 1

A 60-year-old man with a history of dilated cardiomyopathy had a cardiac resynchronization therapy defibrillator (CRT-D) implanted in 2009. Recently, in the context of a newly diagnosed colorectal cancer, he underwent a diverting ileostomy, which led to the development of short bowel syndrome, requiring multiple surgical interventions and hospital admissions. For the administration of chemotherapy, TICVC was placed. Within a year, suspected TICVC infection prompted surgical removal, but the device fractured during the procedure, allowing for removal of only the port-a-Cath reservoir, leaving the distal catheter unretrievable despite multiple attempts (Figure 1); a new right femoral TICVC was subsequently implanted. Six months later, the patient developed a wasting syndrome accompanied by fever. Blood cultures were positive for methicillin-resistant *Staphylococcus aureus*. A transesophageal echocardiogram (TEE) revealed CRT-D lead-associated infective endocarditis. In addition to initiating antibiotic therapy with rifampicin and daptomycin, percutaneous extraction of all the intravascular devices was performed. Lead extraction was carried out using the PISA approach via the left axillary vein, and complete removal of the three leads was achieved. As the right femoral access was required for removal of the retained catheter—and considering the associated infectious risk—the right femoral TICVC was removed. Finally, using a snare introduced through the right femoral vein, the retained right subclavian catheter fragment was successfully retrieved. The procedure was uneventful, with no immediate post-procedural complications. The patient was transferred back to the referring hospital the following day to complete the planned six-week course of intravenous antibiotics for active device-related infection.

**Figure 1:** Chest radiography of the distal portion of the abandoned central venous catheter (yellow arrow) and the CRT-D with a double-coil defibrillation lead.

## Patient 2

A 58-year-old woman with a medical history of right breast cancer diagnosed in 2009 underwent neoadjuvant and adjuvant chemotherapy, for which a left subclavian TICVC was placed in 2010. The patient remained under oncologic follow-up for more than ten years, with no evidence of recurrence. During this period, a malfunction of the device was detected, and it was not possible to carry out the correct lumen catheter washing, which implies an exponential increase of embolic phenomena, secondary to the pro-coagulant environment related to the catheter fibrin adhesion.^6^ For this reason, an attempt was made by interventional radiology to remove the non-functional TICVC, without success. She was then referred to the cardiology department for elective percutaneous extraction of the central venous device (Figure 2). During the procedure, the proximal portion of the catheter was surgically exposed and dissected at the generator pocket on the left side. A guidewire was then introduced through the patent lumen of the device, and using the PISA approach, complete extraction of the catheter was successfully achieved, with clinical and complete radiological success. The patient's hospitalization was uneventful, and she was discharged home the following day.

**Figure 2:** Chest radiography of the TICVC (blue arrow).

## Patient 3

A 63-year-old woman with a history of hypothyroidism, depression, and breast cancer, who had been submitted to right mastectomy six years before, followed by six chemotherapy cycles and hormone therapy, and, in this context, had a TICVC. In the following years, total remission of the pathology was documented, leaving the device unused, despite requiring repeated maintenance to avoid complications. However, during this period, a malfunction of the TICVC was noted. Given the absence of any ongoing indication for its use, a simple traction attempt was made to achieve full device extraction. However, only the Port-a-Cath reservoir could be removed, while the catheter remained in the same place. For this reason, the patient was referred for the extraction of the distal portion of the abandoned catheter in our centre. Through the proximal portion of the catheter, localized in the previously removed Port-a-Cath, and using the dilation sheaths with the PISA technique, the catheter was completely removed (Figures 3 and 4). No complications were registered, and the patient was discharged the day after the procedure.

**Figure 3:** Chest radiography of a TICVC with a guidewire inside, for the removal of the device using the PISA technique.

**Figure 4:** Images of the extraction procedure of the TICVC using the PISA technique.

## Patient 4

A 75-year-old man with a history of transverse colon adenocarcinoma underwent surgical resection one-year prior, followed by eight cycles of adjuvant chemotherapy, for which a TICVC had been placed.

At the 10^th^ month of follow-up, hepatic metastases were identified, and the patient subsequently underwent a right hepatectomy. The postoperative period was complicated by several events, including infectious endocarditis, with methicillin-sensitive *Staphylococcus aureus* isolated in blood cultures. TEE confirmed the presence of vegetation, and the patient was treated with intravenous flucloxacillin for four weeks, with clinical improvement.

One month later, the patient was readmitted with septic shock secondary to *Klebsiella pneumoniae* infection and was treated with targeted antibiotic therapy. Two days after discharge, he developed a new febrile episode. A new TEE was performed, showing no evidence of vegetations.

Considering the recurrent infectious episodes and the suspected infectious focus at the TICVC, the patient was referred for device extraction. The procedure was performed using the PISA technique without complications. During the six-month follow-up, no further infectious episodes were reported.

## Discussion

The removal of long-term TICVCs is indicated when these devices are no longer needed, or when complications or malfunctions occur. In cases of suspected systemic infection, TICVC-related infection must always be considered, and if confirmed, complete device removal is essential.^5,7^

The standard surgical technique for TICVC removal involves an incision over the reservoir, detachment of the catheter, gentle traction for extraction, and final wound closure. Although complications during removal are uncommon, prolonged indwelling time is associated with increased technical difficulty due to the development of a fibrin sheath around the catheter, leading to adhesion to the venous tunica intima. This occurs in approximately 0.3–7.4% of cases and is associated with a higher likelihood of requiring additional interventions.^8^ Interventional radiology offers a stepwise strategy for the removal of adherent port catheters, consisting of three increasingly complex techniques: guidewire support, antegrade coaxial separation, and retrograde coaxial separation.^9^

Previous studies have shown a statistically significant association between indwelling durations exceeding 40 months and difficult removals.^10^ Based on current evidence, several recommendations have been proposed for the safe management of TICVCs: ideally, these devices should be removed promptly after completion of treatment and no later than five years post-implantation, which may reduce the risk of intravascular adhesion and fibrotic encapsulation. If resistance is encountered during attempted extraction, the procedure must be stopped immediately. Forceful traction increases the risk of catheter fracture, vascular avulsion, cardiac tamponade, haemothorax, and haemorrhagic shock.^8^

Among the various approaches for device extraction, the PISA technique provides a safe and effective alternative, particularly in cases where conventional extraction methods are unsuccessful. At our centre, the PISA technique is the preferred method for device removal due to several reasons, including established operator expertise, enhanced control and tactile feedback during catheter manipulation, procedural safety, and cost-effectiveness compared with other extraction techniques. Importantly, this approach also allows the procedure to be safely interrupted at any stage if the risks outweigh the potential benefits—a key consideration in selected non-infectious indications.^5,11–13^ This approach employs polypropylene sheaths (Cook Medical^®^) and polyamide sheaths (FIAB^®^), selecting the sheath whose inner diameter most closely matches that of the catheter to promote dilation and disruption of fibrotic adhesions. Controlled torque is applied using a torque device handle (PIN VISE – Cook Medical^®^ or MGB – FIAB^®^) to advance the mechanical sheath by alternately rotating it clockwise and counterclockwise while maintaining steady traction on the silk ties, thereby releasing the lead from the surrounding fibrotic adhesions. This rotational advancement facilitates dislodgement of the lead tip, allowing extraction without the need for additional extraction techniques.^5,11–16^

A fundamental prerequisite for this technique is that the proximal end of the catheter must be accessible at the vascular entry site; otherwise, it cannot be performed. This was the case for patient 1, in whom access to the proximal end was not possible, necessitating the use of a snare for extraction. In our case series, the use of the PISA technique for TICVC extraction was both clinically and radiologically successful, even following previous unsuccessful surgical attempts.

To our knowledge, this is the first report describing the use of the PISA technique specifically for TICVC extraction. The absence of complications and the high success rate observed suggest this method may be a valuable option in selected patients, particularly those with a high risk of infection or embolic events. As the use of long-term venous access devices continues to grow, adapting established extraction techniques - like the PISA approach - to these catheters may contribute to improved outcomes in complex cases. However, larger studies will be required to confirm these findings.

## Data Availability

The underlying content of the research text is contained within the manuscript.
